# Embryonic stem cell preconditioned microenvironment suppresses tumorigenic properties in breast cancer

**DOI:** 10.1186/s13287-016-0360-x

**Published:** 2016-07-27

**Authors:** Ningning He, Guowei Feng, Yang Li, Yang Xu, Xiaoyan Xie, Hui Wang, Yuebing Wang, Lailiang Ou, Xuetao Pei, Na Liu, Zongjin Li

**Affiliations:** 1 School of Medicine, Nankai University, 94 Weijin Road, Tianjin, 300071 People’s Republic of China; 2 Key Laboratory of Bioactive Materials, Ministry of Education, College of Life Sciences, Nankai University, Tianjin, People’s Republic of China; 3 Institute of Radiation Medicine, Academy of Medical Science and Peking Union Medical College, Tianjin, People’s Republic of China; 4 Department of Genitourinary Oncology, Tianjin Medical University Cancer Institute and Hospital, National Clinical Research Center for Cancer, Key Laboratory of Cancer Prevention and Therapy, Tianjin, 300060 People’s Republic of China; 5 Stem Cells and Regenerative Medicine Laboratory, Beijing Institute of Transfusion Medicine, Beijing, People’s Republic of China; 6 Department of Radiation Oncology, Tianjin Union Medical Center, Nankai University Affiliated Hospital, Tianjin, People’s Republic of China

**Keywords:** Breast cancer cells, Embryonic stem cells, Microenvironment, Stat3 signaling, Imaging

## Abstract

**Background:**

Microenvironment is being increasingly recognized as a critical determinant in tumor progression and metastasis. However, the appropriate regulatory mechanism to maintain the normal balance between differentiation and self-renewal of the cancer cell in microenvironment is not well known.

**Methods:**

4T1 breast cancer cells were treated with embryonic stem (ES) cell conditioned medium which was collected from mouse ES cells. Inhibition of tumor cell growth was based on the reduction of cell proliferation and viability, and inhibition of aggressive properties of tumor cells were examined using the wound-healing and mammosphere assays. The expression of stem cell-associated genes was detected by quantitative RT-PCR.

**Results:**

We used a real-time imaging system to investigate the effect of the mouse ES cell microenvironment on aggressive breast cancer cells in vitro and in vivo. Exposure of breast cancer cells in mouse ES cell conditioned medium resulted in inhibition of growth, migration, metastasis, and angiogenesis of cancer cells. For many tumors, aggressive properties were tightly related to Stat3 signaling activation. We specifically discovered that the ES cell microenvironment sufficiently suppressed Stat3 signaling pathway activation in aggressive tumor cells, leading to a reduction in tumorigenesis and invasiveness.

**Conclusions:**

We identified important functions of Stat3 and their implications for antitumor effects of ES cell conditioned medium. Some factors secreted by ES cells could efficiently suppress Stat3 pathway activation in breast cancer cells, and were then involved in cancer cell growth, survival, invasion, and migration. This study may act as a platform to understand tumor cell plasticity and may offer new therapeutic strategies to inhibit breast cancer progression.

**Electronic supplementary material:**

The online version of this article (doi:10.1186/s13287-016-0360-x) contains supplementary material, which is available to authorized users.

## Background

During embryonic development, the extracellular matrix plays a critical role in normal development and in regulating stem cell differentiation into various lineages, as well as in cell migration and proliferation [1]. The complex relationship between stem cells and their environment plays a crucial role in cell fate decision [2]. Cancer is a complicated disease. Cancer cells also interact with their surrounding microenvironment. The cancer cell microenvironment is constituted of stromata, soluble factors, and cell–cell interactions [3, 4]. The cancer microenvironment affects cancer cell proliferation and growth directly [5]. The microenvironment is being increasingly recognized as a critical component in tumor progression and metastasis. As a malignant cancer, breast cancer can break away from the main malignant tumor and metastasize to other parts of the body by passing through the blood and lymphatic system, so-called cancer metastasis [4, 6]. The microenvironment of breast cancer cells plays a critical role in tumorigenesis and metastasis, which are closely related to activation of the signal transducer activator of transcription 3 (Stat3) signaling pathway. Stat3 is an oncogene and constitutively activated Stat3 has been found in many types of cancer, including breast cancer. Recent studies have suggested that Stat3 activation is important for the tumorigenic ability of cancer stem cells in breast cancer [7, 8].

Some properties of cancer cells are similar to normal stem cells, such as high proliferation, transcriptome expression, signaling transduction, and self-renewal. Subpopulations of cancer cells with extremely high tumorigenic potential, also termed cancer stem cells (CSC) or stem-like cancer cells, have been isolated from cancer patients with a variety of tumor types [9–16]. Cancer stem cells in breast cancer cells are capable of self-renewal and have the ability to initiate breast tumor progression [15, 17, 18]. But different from normal stem cells, the proliferation of cancer cells is out of control and loss of differentiation plasticity. As already mentioned, microenvironment is very important for cell proliferation and properties. The difference between cancer cells and normal stem cells might be caused by the different microenvironments surrounding them. Recent findings also indicated a tumor-suppressive effect of an embryonic microenvironment on human metastatic melanoma cells [19]. Besides the embryonic microenvironment, the embryonic stem (ES) cell microenvironment has also been shown to alter behaviors of a variety of cancer cells, such as invasiveness and tumorigenicity [2, 5]. Furthermore, several groups reported that the human ES cell microenvironment might suppress melanoma tumor cells by secretion of Lefty into the matrix [20].

Although several publications have reported the ability of embryonic and ES cell microenvironments to reprogram cancer cells toward a benign phenotype, the mechanism by which the microenvironment regulates cancer cell proliferation and tumorigenicity has not been elucidated clearly. Understanding the tumor-suppressive effect of the ES cell microenvironment may further offer new therapeutic strategies to inhibit tumor progression. In this context, we used ES cell conditioned medium and an imaging approach to address whether aggressive cancer cells could respond to mouse embryonic stem cell (mES) conditioned medium, and to investigate the possible underlying mechanisms. Using this approach, we found that exposure of breast cancer cells to the ES cell microenvironment resulted in reduced invasive potential, which might be mediated by Stat3 signaling inactivation.

## Methods

### Cell culture

Mouse breast cancer cell line 4T1 was purchased from ATCC (Manassas, VA, USA) and was grown in RPMI 1640 medium supplemented with 10 % FBS, 1 % penicillin–streptomycin solution (Gibco), and 1 % MEM nonessential amino acid solution (Gibco). The murine ES cell line J1 (SCRC-1010™; ATCC) of the SV129 strain was cultured on mouse embryonic fibroblast feeder layers inactivated by 10 μg/ml mitomycin C in DMEM containing 15 % ES-qualified FBS (HyClone), 1000 units/ml of LIF (Millipore), 0.1 mM β-mercaptoethanol, 0.1 mM nonessential amino acids (Gibco), and 100 U/l penicillin/streptomycin (Gibco). To perform the experiments using imaging, 4T1 cells were transduced with a lentiviral vector carrying a ubiquitin promoter driving firefly luciferase (Fluc) and enhanced green fluorescence protein (eGFP) followed by a Stat3 specific-binding promoter driving renilla luciferase (Rluc) reporter gene responding to activated Stat3. For co-culture assay, 4T1 cells and ES cells with the red fluorescence protein (RFP) reporter gene were constructed.

### Collection of conditioned medium

The mES cells, 4T1 cells, or SNL cells (purchased from ATCC) were cultured to 40 % confluence with each usual culture medium respectively, and the medium for ES cells and SNL cells was changed to 7 ml DMEM and 5 % FBS per T25 flask (Corning). The medium for 4T1 cells was changed to 7 ml RPMI 1640 and 5 % FBS per T25 flask (Corning). Forty-eight hours later, the supernatants were harvested and stored at –80 °C. As already described, ES cells can maintain the self-renewal and pluripotency properties in vitro, but need fibroblast feeder cells for support. In our study, SNL cells – an immortalized fibroblast cell line – were used as feeder layers to support the growth and pluripotency of mES cell in-vitro cultures. To exclude the effect made by SNL cells when the ES conditioned medium (ES-SNL-CM) was collected from the ES cells cultured on SNL cells, we used the SNL conditioned medium (SNL-CM) as a parallel control. If ES conditioned medium (ES-CM) was collected from ES cells cultured on gelatin without feeder cells, DMEM medium was used as its control. 4T1 cell conditioned medium (4T1-CM) severed as the mock control group.

### Morphological observation

4T1 cells were seeded in six-well plates (Corning) at a density of 2.5 × 10^5^ per well. After the cells attached, the medium was changed to 4T1-CM and ES-CM mixed with the same amount of RPMI 1640 (supplemented with 10 % FBS) respectively. The medium in another control group was changed to basal DMEM and 5 % FBS mixed with the same amount of RPMI 1640 (supplemented with 10 % FBS). The bright micrograph of each group was taken 48 h after the medium was changed.

### Cell proliferation and viable assays

4T1 cells were seeded in 12-well plates (Corning) at a density of 3 × 10^4^ per well. After the cells attached, the medium was changed to different CMs and mixed with RPMI 1640 medium (supplemented with 10 % FBS). Five fields per well were chosen randomly and marked. Cells in the chosen fields were counted at 0, 1, 2, and 3 days, respectively. Viable cells were assessed by trypan blue dye exclusion assay at intervals and population doublings were calculated.

### Optical imaging

Optical imaging was performed using the IVIS200 Imaging System (Xenogen Corporation, Hopkinton, MA, USA). Bioluminescence imaging of the fate of transplanted cells in living mice was done during 21 days. Fluc imaging of 4T1 cells was used for assessing tumor development and Rluc imaging was used for assessing the level of activated Stat3 expression. d-Luciferin (150 mg/kg; Biosynth International, Naperville, IL, USA) was intraperitoneally injected into mice for evaluating Fluc expression, and each mouse was imaged for 1–10 min. Coelenterazine (2.5 mg/kg; NanoLight Technology, Pinetop, AZ, USA) was injected intravenously into mice to assess Rluc expression. After injection of coelenterazine, mice were immediately imaged for 2 min [21, 22].

### Real-time PCR

To assess the mRNA expression levels of the genes, total RNA was extracted from 4T1 cells treated with different CMs with TRIzol reagent according to instructions supplied by the manufacturer. Then 2 μg of total RNA was used for the first-strand cDNA template synthesis (TIANscriptRT kit; TIANGEN Biotech) and real-time PCR was performed with the Opticon® System (Bio-Rad) using the TransStart Green qPCR Super Mix Kit (TransGen Biotech). Quantitative RT-PCR was estimated using the 2^–ΔΔct^ method. Primers are presented in Additional file 1: Table S1.

### Western blotting

To explore protein expression, western blotting was performed after the samples were harvested. Each group of 4T1 cells was lysed on ice in RIPA buffer (CW2333; cwbiotech) plus protease inhibitor cocktail and phosphatase inhibitors. Equivalent amounts of protein (BCA protein assay) from each sample were used for immunoblot analysis as described previously [21]. Proteins were examined with specific antibodies against Sox2 (Santa Cruz Biotechnology), Stat3 (Santa Cruz Biotechnology), p-Stat3 (Abcam), Oct4 (Santa Cruz Biotechnology), β-catenin (Santa Cruz Biotechnology), Nanog (Bethyl), and β-actin (Santa Cruz Biotechnology).

### Immunofluorescence staining

For immunofluorescence staining, rat anti-mouse CD31 antibody (BD Bioscience, Bedford, MA, USA) was used for determining angiogenesis in tumors. Specific antibodies against Stat3 (Santa Cruz Biotechnology) and p-Stat3 (Abcam) were used for detecting the expression level of activated Stat3 in tumors. Alexa Fluor 594-labeled secondary antibody (Invitrogen) was used for detection.

### Wound-healing assay

4T1 cells were seeded in six-well plates at a density of 2 × 10^5^ per well. After the cells attached, the medium was changed to 4T1-CM, DMEM, or ES-CM mixed with the same amount of RPMI 1640 (supplemented with 10 % FBS). The medium was removed when the cells were cultured to 90 % confluence, and then three separate wounds were scratched with sterile 20 μl tips (Corning). Cells were rinsed with PBS, and 2 ml RPMI 1640 supplemented with 2 % FBS per well was added. Photographs were taken at 0, 24, and 48 h, respectively [23]. The percentage of wound healed was calculated using the formula:

$$ \left[1\hbox{--} \left( final\  area/ initial\  area\right)\right]\times 100\%. $$

### In-vivo tumor genesis test

A group of female BALB/c mice (*n* = 6 mice/group), 8–12 weeks old, were injected with 2 × 10^5^ 4T1 (Fluc/GFP-pStat3/Rluc) cells after treatment with 4T1-CM for 48 h into the left of the fourth pair of mammary fat pads (day 0) [24]. Equal numbers of 4T1 (Fluc/GFP-pStat3/Rluc) cells after treatment with ES-CM for 48 h were injected into the right of the fourth pair mammary fat pads for detecting the tumor growth and the expression level of activated Stat3 by imaging. Mice were sacrificed at 21 days after tumor cell challenge. All animal procedures were approved by the animal Ethic Committee of Nankai University (Tianjin, China) and followed the Guide for the Care and Use of Laboratory Animals established by Nankai University.

### Mammosphere assay

To generate mammospheres, cells were grown in serum-free, growth factor-enriched conditions in low attachment plates [25–27]. 4T1 cells after treatment with 4T1-CM and ES-CM for 48 h were grown in six-well ultralow-attachment plates in serum-free RPMI 1640 supplemented with 20 ng/ml bFGF, 20 ng/ml hEGF, and B27 (all from Invitrogen). Cells were plated at 8000 cells/per well. Suspension cultures were incubated for 4 days. Colonies were counted under the microscope.

### Direct co-culture assay

4T1 breast cancer cells were co-cultured with mES cells. For co-culture with direct cell–cell contact, 2 × 10^5^ 4T1 (Fluc/GFP-pStat3/Rluc) cells mixed with 2 × 10^5^ 4T1 (RFP) cells were seeded in one well of a six-well plate as a control group. For the experimental group, 4T1 (Fluc/GFP-pStat3/Rluc) cells were mixed with the same amount of mES (RFP) cells. Fluorescent photographs were taken at 24, 48 and 72 h.

### Statistical analysis

Statistical analysis was performed using Graphpad Prism 5.0 software (Graphpad Software Inc.). Two-way repeated-measures ANOVA and two-tailed Student’s *t* test were used. *P* < 0.05 was considered statistically significant. All experiments were performed at least three times with triplicate samples.

## Results

### Generation of labeled tumor cells for real-time imaging

**Fig. 1:**
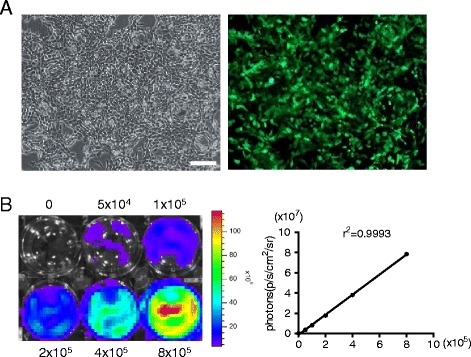
Transduction of 4T1 cells with Fluc/GFP and pStat3/Rluc reporter genes. **a** Transduced 4T1 cells are strongly positive for eGFP on fluorescence microscopy. **b** Ex-vivo imaging analysis of stably transduced 4T1 cells shows a strong correlation between cell numbers and Fluc reporter gene activity. Scale bar represents 100 μm

In order to investigate the effect of the ES cell microenvironment on cancer cells, we planned to treat breast cancer cells with ES-CM. The 4T1 breast cancer cell line was used in this study. In order to track the transplanted cancer cells in vivo using imaging analysis, 4T1 cells were transduced with a self-inactivating lentiviral vector carrying a ubiquitin promoter driving firefly luciferase (Fluc) and enhanced green fluorescence protein (eGFP) followed by a seven-repeat Stat3-binding sequence (enhancer) and minimal TA (promoter) driving renilla luciferase (Rluc) reporter genes (4T1-Fluc/GFP-pStat3/Rluc). The fluorescence imaging results indicated that eGFP was robustly expressed in 4T1 cells (Fig. 1a). FACS analysis revealed that the percentage of GFP-positive cells was more than 95 % (data not shown). We next investigated the correlation between the cell proliferation and firefly signal intensity. A strong correlation (*r*^*2*^ = 0.99) was observed between Fluc activity and 4T1 cell numbers in vitro using the Xenogen IVIS system, which revealed the availability of assessing tumor growth in vivo by analyzing firefly signal intensity (Fig. 1b). The expression of Rluc was under the control of Stat3 activation. The signaling intensity of Rluc therefore indicated the Stat3 signaling activation in 4T1 cancer cells.

### Effect of microenvironment of mES cells on the proliferation of aggressive breast cancer cells

**Fig. 2:**
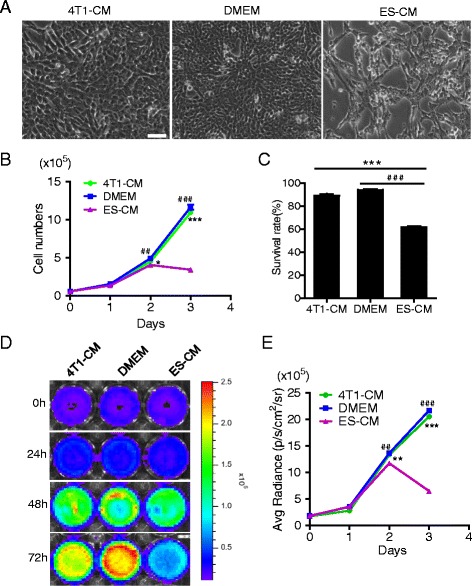
Effect of the microenvironment of ES cells on the proliferation of aggressive breast cancer cells. **a** Microscopy showed a morphological change in cells treated with ES-CM. Scale bar represents 100 μm. **b** Growth curves made by counting cell numbers every day revealed a decreased proliferation rate of cells treated with ES-CM. *P < 0.05 vs 4T1-CM, ^##^ *P* < 0.01 vs DMEM, ***P < 0.001vs 4T1-CM, ^###^ *P* < 0.001 vs DMEM, *n* = 3. **c** Trypan blue cell viability assay showed a low survival rate. ***P < 0.001 compared with controls, *n* = 3. **d** Fluc imaging showed decreasing bioluminescence signals at 24, 48, and 72 h after culturing cells with ES-CM. **e** Quantitative analysis of imaging signals. The signal activity showed the suppressed effect growth of ES-CM group. **P < 0.01 vs 4T1-CM, ^##^ *P* < 0.01 vs DMEM, ***P < 0.001 vs 4T1-CM, ^###^ *P* < 0.001 vs DMEM, *n* = 3. *CM* conditioned medium, *DMEM* Dulbecco’s modified Eagle medium, *ES* embryonic stem

To investigate the effect of the ES cell microenvironment on cancer cells, 4T1 cells were treated with J1 ES-CM. Tightly aggregated and uniformly round ES cell colonies were observed in J1 cells by phase-contrast microscope. J1 cells have a high expression level of Ssea1. Alkaline phosphatase is also positive in J1 cells (Additional file 1: Figure S1A). These data indicated that J1 cells were sustained in a pluripotent state and can be used in the next study. J1 cells were cultured to 40 % confluence with usual culture medium on gelatin without feeder cells, and the medium was then changed to DMEM medium with 5 % FBS. ES-CM was collected from J1 cells 48 h later. 4T1 cancer cells were treated with ES-CM and control medium (DMEM or 4T1-CM) respectively. 4T1 cells treated with ES-CM lost their normal phenotype 48 h later, compared with their controls. The number of cells was sharply reduced (Fig. 2a).

Then we examined the proliferation of 4T1 cells treated with different CMs and found that the 4T1 cells treated with ES-CM grew slowly compared with controls. As shown in Fig. 2b, at day 3 the proliferation of 4T1 cells was obviously inhibited by ES-CM compared with the other two control groups, whereas little or no difference was observed in 4T1 cells treated with 4T1-CM and DMEM medium. Bioluminescence imaging of Fluc was further performed to identify the cell number of 4T1 cells in each group. The Fluc activity was decreased in cells treated with ES-CM compared with the two control groups (Fig. 2d). Quantitative analysis showed that the Fluc signal intensity of 4T1 cells treated with ES-CM was less than half that of the control groups (Fig. 2e), consistent with the cell number counting test. Trypan blue staining assay was used to detect the cell survival rate, which showed a decreased survival rate in cells treated with ES-CM (Fig. 2c).

From these results, we can conclude that ES cell preconditioned medium efficiently inhibited cancer cell proliferation. Next, we further investigated the effect of the ES cell microenvironment on cancer cells using a direct co-culture model. In order to distinct the two co-culture cells easily, we tested 4T1 cells and J1 cells with the RFP reporter gene. RFP was robustly expressed in 4T1 cells and mES cells respectively (Additional file 1: Figure S1B, C), which can be distinguished from GFP-positive 4T1 cells in the co-culture system. J1 ES cells and 4T1 cells with RFP were cultured with 4T1 cells (with GFP reporter gene). The 4T1 cells, which were co-cultured with ES cells, were reduced 72 h later (Additional file 1: Figure S2). These results further indicated that the ES cell microenvironment could efficiently inhibit the proliferation of cancer cells.

### Microenvironment of ES cells inhibited Stat3 signaling activation in 4T1 cells in vitro

Stat3 regulates many critical functions in normal and malignant tissues, such as proliferation, differentiation, survival, angiogenesis, and immune function [28]. Two Stat3 signaling target genes, *c-Myc* and *Klf4*, play critical roles in cancer stem cell self-renewal [29–34]. Also, Stat3 controls cancer cell fate and interaction with the microenvironment; it plays important roles in the maintenance of cancer stem cell populations, switching between epithelial and mesenchymal phenotypes that precede metastasis, and tumor angiogenesis [35].

**Fig. 3:**
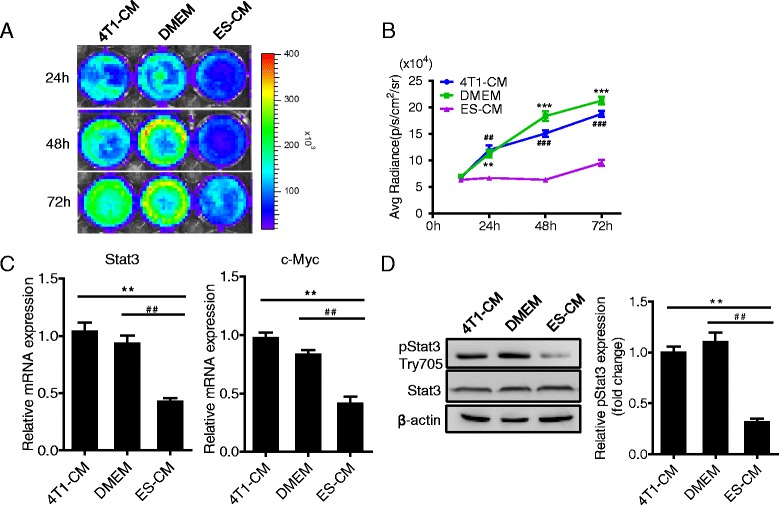
ES-CM inhibited Stat3 signaling pathway in 4T1 cells. **a** Rluc imaging of activated Stat3 in vitro controlled by 4T1-CM and DMEM. **b** Quantitative analysis of imaging signals. The signal activity showed the suppressed effect of ES-CM group. **P < 0.01 vs 4T1-CM, ^##^ *P* < 0.01 vs DMEM, ***P < 0.001 vs 4T1-CM, ^###^ *P* < 0.001 vs DMEM, *n* = 3. **c** PCR analysis of Stat3 signaling pathway-related gene expression. **P < 0.01 vs 4T1-CM, ^##^ *P* < 0.01 vs DMEM, *n* = 3. **d** ES-CM decreased the phosphorylation of Stat3 in 4T1 cells. Representative western blots showing the level of pStat3 and total Stat3. Data are presented as mean ± SD. **P < 0.01 vs 4T1-CM, ^##^ *P* < 0.01 vs DMEM, *n* = 3. See also Additional file 1: Figure S3. *CM* conditioned medium, *DMEM* Dulbecco’s modified Eagle medium, *ES* embryonic stem

To explore the activation of Stat3 signaling pathway in 4T1 cells in the ES cell microenvironment, the Rluc imaging was evaluated in the experiments, which indicated the level of activated Stat3. After treatment with different CMs for 24, 48 and 72 h, the Rluc activity was significantly decreased in the ES-CM group by analyzing the renilla signal intensity (Fig. 3a, b). We examined the gene expression level of Stat3 signaling pathway-related genes *c-Myc* and *Stat3* which were obviously downregulated in the ES-CM group compared with the control groups (Fig. 3c). Consistent with this, the phosphorylated Try-705 Stat3 was obviously lower in 4T1 cells treated with ES-CM, which indicated that ES-CM suppressed Stat3 signaling activation significantly (Fig. 3d).

In all of these experiments we used ES-CM obtained from ES cells without feeder cells. As is well known, J1 ES cells can sustain their undifferentiated state on feeder cells (SNL cells). We next compared the effect of ES-SNL-CM and SNL-CM on cancer cells. In this experiment, ES-CM was collected directly from ES cells cultured on feeder cells for 48 h after changing to basic DMEM medium, and the control medium was collected from only feeder cells (SNL cells). Consistent with the no-feeder cell culture system, the Stat3 signaling pathway was inhibited in cancer cells treated with preconditioned medium only when ES cells were present (Additional file 1: Figure S3).

### Microenvironment of ES cells inhibited Stat3 signaling activation in 4T1 cells in vivo

**Fig. 4:**
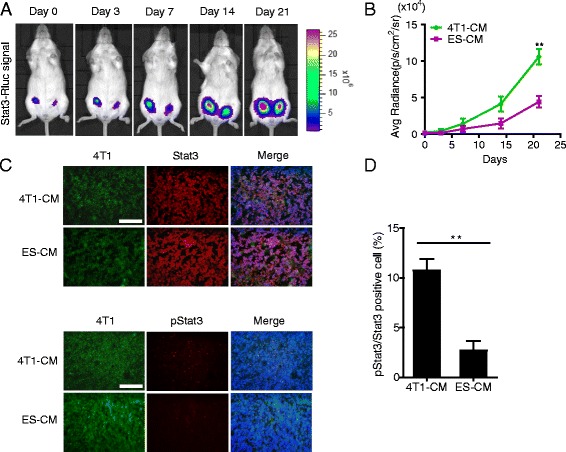
Inhibitory effect of ES-CM on Stat3 signaling in vivo*.* **a** Renilla luciferase (*Rluc*) imaging of activated Stat3. Representative animals injected with 2 × 10^5^ 4T1 (Fluc/GFP-pStat3/Rluc) cells after treatment with 4T1-CM and ES-CM for 48 h into the left and right of the fourth pair of mammary fat pads respectively. **b** Quantitative analysis of Rluc signal indicated decreased p-Stat3 level. **P < 0.01 vs 4T1-CM, *n* = 5. **c** Representative examples of the expression of Stat3 and pStat3 of tumor tissue by immunofluorescence staining. Scale bar represents 100 μm. **d** Quantitative analysis of pStat3 to total Stat3 expression. **P < 0.01 vs 4T1-CM, *n* = 3. *CM* conditioned medium, *ES* embryonic stem

To confirm that ES-CM which we defined as the ES cell microenvironment could also inhibit the Stat3 activation in vivo, equal numbers of 4T1 cells (Fluc/GFP-pStat3/Rluc), which were treated with 4T1-CM and ES-CM respectively for 48 h, were implanted subcutaneously into female BALB/c mice and allowed to form tumors for 21 days. 4T1 cells treated with 4T1-CM and ES-CM were injected into the left and right mammary fat pad respectively. Then we performed Rluc imaging to monitor the level of activated Stat3 in vivo using the IVIS imaging system. We observed less Rluc activity signal in the ES-CM-treated group (Fig. 4a, b). We further investigated the pStat3 level in the tumor formed by 4T1 cells (treated with different media) in mice. Immunofluorescence staining of Stat3 and phosphorylated Try-705 Stat3 of the tumors formed in mice confirmed these results (Fig. 4c, d). These data demonstrated that ES-CM significantly inhibited the Stat3 signaling pathway no matter whether in vitro or in vivo*.*

### Antitumor effect of ES cell microenvironment in vivo

**Fig. 5:**
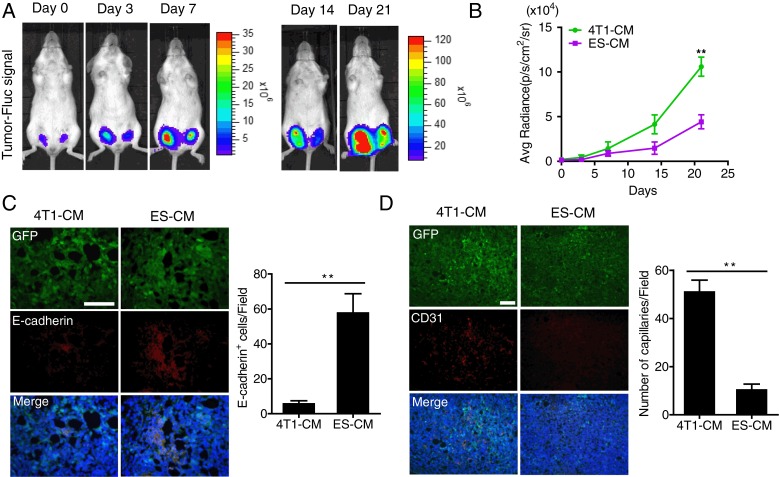
Inhibitory effect of ES-CM on tumor growth *in vivo.* **a** Firefly luciferase (*Fluc*) imaging of tumor progression. Representative animals injected with 2 × 10^5^ 4T1 (Fluc/GFP-pStat3/Rluc) cells after treatment with 4T1-CM and ES-CM for 48 h into the left and right of the fourth pair of mammary fat pads respectively. **b** Quantitative analysis of Fluc signal. **P < 0.01 vs 4T1-CM, *n* = 5. **c, d** Representative immunofluorescence staining of E-cadherin and CD31 of tumor tissue demonstrated a resistance of EMT progress and decreased tumor angiogenesis ability. Percentages of E-cadherin and CD31-positive cells are shown. Scale bar represents 100 μm. **P < 0.01 vs 4T1-CM, *n* = 3. See also Additional file 1: Figure S4. *CM* conditioned medium, *ES* embryonic stem, *GFP* green fluorescent protein

To determine whether the reduced expression of Stat3 made the contribution of inhibiting tumor growth, Fluc activity was measured to monitor the tumor progression in vivo. Signal intensity indicates the tumor size. From the results we can see that the signal increased more slowly in the ES-CM-treated group than in the 4T1-CM-treated group (Fig. 5a, b). Consistent with this, the weight and volume were also significantly decreased in the ES-CM-treated tumor group (Additional file 1: Figure S4A, B). These results revealed that some factors secreted by ES cells had an inhibitory effect on 4T1 tumor growth by repressing the Stat3 signaling pathway. This experiment demonstrated a reduced malignancy of ES-CM-treated 4T1 and prompted us to further explore the mechanism attributing to this phenomenon in vitro.

### Effects of ES cell microenvironment on breast cancer cell migration, metastasis, and angiogenesis

**Fig. 6:**
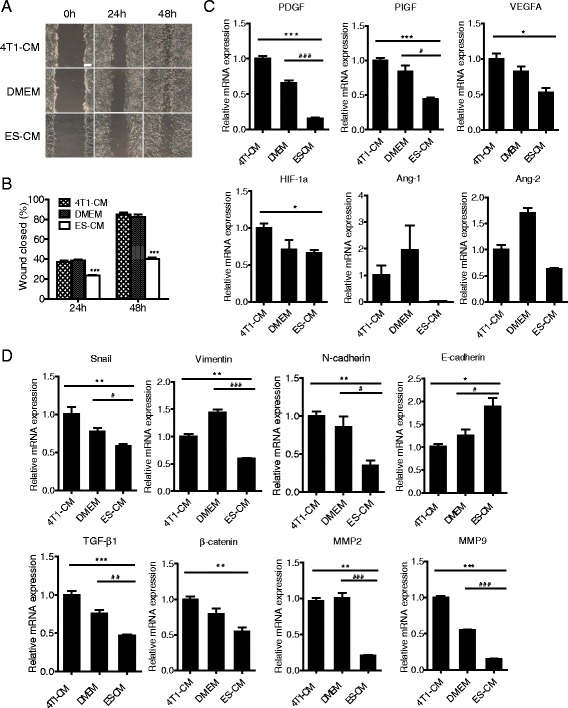
Effects of the ES cell microenvironment on breast cancer cell migration, metastasis, and angiogenesis. **a** Representative photographs of wound-healing assay of 4T1 cells treated with 4T1-CM, DMEM, and ES-CM. Scale bar represents 100 μm. **b** Wound healing area per field was decreased after being treated with ES-CM. ***P < 0.001 compared with controls, *n* = 3. **c** Analysis of angiogenic factors expression in 4T1 cells treated with different CMs. *P < 0.05 vs 4T1-CM, ***P < 0.001 vs 4T1-CM, ^#^ *P* < 0.05 vs DMEM, ^###^ *P* < 0.001 vs DMEM, *n* = 3. **d** Expression of metastasis and EMT-related genes determined by RT-PCR. All experiments were performed at least in triplicate and data are presented as mean ± SD. *P < 0.05 vs 4T1-CM, **P < 0.01 vs 4T1-CM, ***P < 0.001 vs 4T1-CM, ^#^ *P* < 0.05 vs DMEM, ^##^ *P* < 0.01 vs DMEM, ^###^ *P* < 0.001 vs DMEM, *n* = 3. See also Additional file 1: Table S1. *CM* conditioned medium, *DMEM* Dulbecco’s modified Eagle medium, *ES* embryonic stem, *MMP* matrix metalloproteinase, *VEGF* vascular endothelial growth factor

**Fig. 7:**
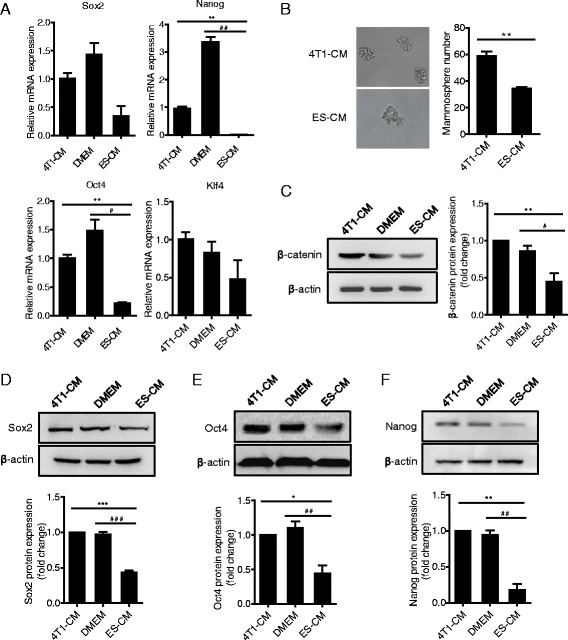
ES-CM weakened 4T1 cancer cell stemness. **a** Real-time RT-PCR analysis of stemness-related gene expression of 4T1 cells in each group treated with CMs for 48 h. **P < 0.01 vs 4T1-CM, ^##^ *P* < 0.01 vs DMEM, *n* = 3. **b** 4T1 cells after treatment with 4T1-CM and ES-CM for 48 h respectively were plated in low-density suspension cultures for sphere formation 4 days after plating. Experiment performed in triplicate. Data presented as mean ± SD. **P < 0.01 vs 4T1-CM. **c–f** Protein expression level of β-catenin **c**, Sox2 **d**, Oct4 **e**, and Nanog **f** in 4T1 cells examined by western blot. *P < 0.05 vs 4T1-CM, ^#^ *P* < 0.05 vs DMEM, **P < 0.01 vs 4T1-CM, ^##^ *P* < 0.01 vs DMEM, ***P < 0.001 vs 4T1-CM, ^###^ *P* < 0.001 vs DMEM, *n* = 3. *CM* conditioned medium, *DMEM* Dulbecco’s modified Eagle medium, *ES* embryonic stem, *MMP* matrix metalloproteinase

Our data showed that cells treated with EC-CM exhibited obvious proliferative inhibition and downregulated Stat3 expression. Stat3 pathway is tightly associated with tumor invasion, metastasis, and angiogenesis in cancers [35, 36]. We then examined the effects of the ES cell microenvironment on the migration, metastasis, and angiogenesis of 4T1 cells. In this regard, we performed a wound scratch test and generated gene expression profiles of aggressive breast cancer cells (4T1 cells) treated with ES-CM and two other control media. After 48 h, a significant difference was observed between ES-CM and control groups. The migration ability of cancer cells was impaired by ES-CM (Fig. 6a, b). Cancer cell migration is tightly related to epithelial–mesenchymal transition (EMT) [37, 38]. Therefore, we then analyzed the expression level of several genes involved in the EMT process. Our results showed that these cells treated with ES-CM decreased expression of mesenchymal markers (such as *vimentin*, *N-cadherin*, *Snail*) and increased expression of epithelial markers (such as *E-cadherin*) related to the EMT process (Fig. 6d). Similarly, the protein expression of E-cadherin was upregulated in tumor tissue formed by cells treated with ES-CM, which indicated a resistance of ES-CM to the EMT progress (Fig. 5c). Furthermore, the invasion-related genes (*Tgf-β1*, *β-Catenin*, *Mmp2*, *Mmp9*) also increased (Fig. 6d). Western blot analysis confirmed that the protein level of β-Catenin was also upregulated (Fig. 7c). All of these results demonstrated that some soluble factors of ES-CM probably impaired tumor metastasis ability through the EMT pathway.

In addition, we also examined the change of tumor angiogenesis treated with different CMs. CD31 is commonly used to evaluate the degree of tumor angiogenesis. Immunofluorescence staining results revealed that microvascular density was significantly decreased in the ES-CM-treated tumor tissue group (Fig. 5d). Besides CD31, other angiogenesis-related gene expression also decreased in the ES-CM group (Fig. 6c), which indicated that ES-CM might inhibit angiogenesis. All of these results showed that ES-CM could inhibit breast cancer cell migration, metastasis, and angiogenesis.

### ES-CM weakened 4T1 cancer cell stemness

**Fig. 8:**
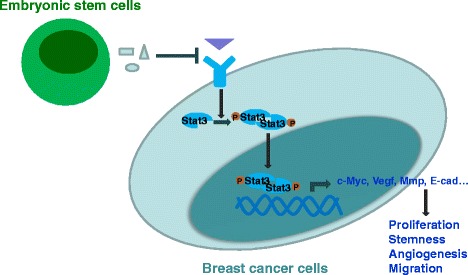
Proposed model for tumor-suppressive effects of ES-CMs. Some factors secreted by ES cells could efficiently suppress Stat3 pathway activation in breast cancer cells. The Stat3 signaling pathway regulates the expression of *E-cadherin*, *Vegf*, c-Myc, and *Mmp*, and is then involved in cancer cell growth, survival, invasion, and migration. *MMP* matrix metalloproteinase, *Stat3* signal transducer and activator of transcription 3, *VEGF* vascular endothelial growth factor

The cancer stem cell hypothesis has gained significant recognition as a descriptor of tumorigenesis [39]. Cancer stem cells have been identified to be the vital cause of cancer recurrence and relapse due to their resistance to chemotherapy and radiotherapy [40]. Therefore we made a hypothesis that ES-CM might inhibit cancer cell stemness, and then effectively suppress cancer cell metastasis and relapse. In order to investigate this hypothesis, we further determined the stemness-associated gene expression in 4T1 cells treated with different CMs. Our data showed that the expression levels of *Sox2*, *Nanog*, *Oct4*, and *Klf4* were decreased in 4T1 cells treated with ES-CM (Fig. 7a). Sox2, Oct4, and Nanog protein levels were next confirmed using western blot analysis, which was significantly decreased in ES-CM-treated 4T1 cells (Fig. 7d–f). Pluripotent-related gene downregulation indicated that stemness of 4T1 cells was weakened. To make a further analysis, we performed a mammosphere formation experiment, which was often used as a method for detecting the tumor-initiating capacity [16]. In the ES-CM-treated group, mammosphere formation efficiency was significantly lower than in the control group (Fig. 7b). These results indicated that ES-CM efficiently weakened 4T1 cancer cell stemness. We speculated that decreased mammosphere formation might be due to some factors in the ES-CM secreted by ES cells. Cells with reduced stemness-associated gene expression are poor for mammosphere formation potential and poor for tumor-initiating capacity. All of these data demonstrated that the malignant activities of 4T1 cells were largely related to activation of the Stat3 signaling pathway which made a significant contribution to the migration, metastasis, angiogenesis, and stemness of 4T1 cells (Fig. 8).

## Discussion

Over the last decade, a variety of imaging technologies have been investigated as tools for cancer diagnosis and monitoring response to cancer therapies [41]. Molecular imaging offers the potential for noninvasive assessment therapeutic responses and real-time monitoring of tumor procession simultaneously [22, 41]. In addition to this, molecular imaging provides the possibility to visualize and monitor cellular and molecular processes, such as metabolism, biosynthesis, angiogenesis, cell proliferation, and apoptosis [42–44]. In this study, we evaluated the effect of ES-CM on cancer procession by dual imaging. 4T1 cancer cells were transduced with a vector carrying a ubiquitin promoter driving Fluc followed by a seven-repeat Stat3-binding sequence (enhancer) and minimal TA (promoter) driving Rluc reporter genes (4T1-Fluc/GFP-pStat3/Rluc). Fluc and Rluc have different substrates, d-luciferin and coelenterazine respectively. Using this bioluminescence imaging system, we can simultaneously track the ES-CM on tumor growth (Fluc) and Stat3 signal pathway activation (Rluc) in vivo and in vitro. This model enabled us to obtain more tangible options for the ES microenvironment on tumor growth and Stat3 signaling pathway activation. Based on this model, we demonstrated that the ES cell microenvironment played a critical antitumor role in vitro and in vivo*.* This may be mediated by the Stat3 pathway.

As already mentioned, certain phenotypic characteristics are shared by ES cells and some aggressive cancer cells, such as unlimited self-renewal and expression of some pluripotent genes (*NANOG*, *OCT4*, *SOX2*) [45]. However, cancer cells lack the appropriate regulatory mechanisms to maintain normal differentiation. This difference might be attributed to the different microenvironments surrounding them. Since the embryonic microenvironment possesses key regulatory cues and signaling molecules that function to maintain and regulate the growth of the stem cell population, we hypothesized that an ES cell microenvironment might be able to influence cancer cells by normalizing their plastic phenotype. Some researchers have revealed that the microenvironment of human ES cells is able to change and reprogram aggressive cancer cells to a less aggressive state. Some mechanisms involved in the phenotypic changes have been proved to associate with the Nodal signaling pathway, which plays a key role in tumor cell plasticity [20, 46]. However, as for mES cells, several other molecular mechanisms might be related directly and/or indirectly to these changes, including the Stat3 signaling pathway. Therefore in this article we investigated in detail of the effect of the ES cell microenvironment on breast tumor progression and metastasis. The results of our study demonstrate that exposure of breast cancer cells to the ES cell microenvironment downregulates Stat3 signaling, associated with a reduction in clonogenicity and tumorigenicity.

Stat3 has been identified with critical importance for maintaining cancer stemness [16]. Stat3 is an oncogene expressed in many cancers including breast cancer, prostate cancer, lung cancer, head cancer, liver cancer, pancreatic cancer, and multiple myeloma [47–49]. Stat3 has also been found to be involved in cancer cell growth, survival, invasion, and migration through regulation of the expression of E-cadherin, VEGF, and MMPs [19, 50]. In addition to these kinds of roles, Jak–Stat3 signaling has recently been demonstrated to have central roles in premetastatic niche formation [51–54]. Evidence is also accumulating for the important roles of Stat3 in breast cancer stem cells [55]. Stat3 ablation leads to decreased tumor cell proliferation and growth [56]. In this study, we identify important functions of Stat3 and their implications in the antitumor effect of ES-CM. We show that the environment created by ES cells has a suppressive effect on 4T1 cells by downregulation of Stat3 in these tumor cells. The Stat3 signaling pathway stimulates cell proliferation and migration/invasion. Using a bioluminescent imaging system, we have shown that inhibiting Stat3 mediated by ES-CM in tumor-bearing mice dramatically decreased both the growth rates and volumes of the tumor. We provided evidence that downregulation of Stat3 was critical for the inhibition of cancer cells in vivo.

Activation of Stat3 is associated with metastasis in many tumors. This association may be attributed to the overexpression of several growth factors, MMP2 and VEGF, which are induced by Stat3 activation and subsequently promote tumor invasion and angiogenesis [57]. Consistent with previous findings, the inhibition of Stat3 in our breast cancer mouse model resulted in a lower metastasis rate. *Mmp2* and *Vegf* expression was downregulated in the ES-CM-treated group. The weight and volume of the tumor formed by 4T1 cells treated with ES-CM were also significantly decreased. Our in-vitro assays confirmed that ES-CM weakened the migration, metastasis, and angiogenesis of 4T1 cells, which may act by inhibiting Stat3. We thus propose that the microenvironment created by ES cells could inhibit the tumor growth possibly through downregulating the Stat3 signal pathway.

It has been commonly agreed that cancer cell behavior largely depends on the tumor microenvironment, which is very complex and consists of cells, growth factors, extracellular matrix, and extracellular vesicles (EVs) [38, 58, 59]. A growing number of studies suggest that ES-CM efficiently suppresses the invasive potential of cancer cells [2, 5]. The human ES cell microenvironment suppresses melanoma tumor cells by secretion of Lefty into the matrix [20]. Our data suggested that some factors secreted by ES cells could efficiently suppress the Stat3 pathway in breast cancer, resulting in a loss of tumorigenicity. Besides these, some other mechanisms might be also involved in the antitumor effect of the ES cell microenvironment. Understanding the plastic phenotype expressed by the aggressive tumor cells in response to their environment is helpful to develop therapeutic strategies in patients with cancer [1, 2]. More detailed future studies are therefore required to illustrate the detailed mechanisms involved in the suppression effect of the ES cell microenvironment on cancer cells.

## Conclusion

Our findings provide evidence that the mouse embryonic microenvironment may contain certain factor(s) that are capable of inhibiting growth, migration, metastasis, and angiogenesis of cancer cells, which may be through the Stat3 signaling pathway and has the potential to reprogram cancer cells into a less invasive phenotype. These results may represent a new cancer treatment strategy and may be used as potential targets in new therapeutic approaches.

## Abbreviations

CM, conditioned medium; DMEM, Dulbecco’s modified Eagle medium; eGFP, enhanced green fluorescence protein; EMT, epithelial–mesenchymal transition; ES, embryonic stem; FBS, fetal bovine serum; MMP, matrix metalloproteinases; PBS, phosphate-buffered saline; RFP, red fluorescence protein; Stat3, signal transducer and activator of transcription 3; VEGF, vascular endothelial growth factor

## Additional file

Additional file 1: Supplementary information contains Table S1 presenting primer sequences used for real-time PCR, Figure S1 showing mES cell characterization and reporter gene cells used in the experiment, Figure S2 showing decreased proliferation of 4T1 cells by direct cell–cell contact of ES cells, Figure S3 showing ES-CM inhibited Stat3 signaling pathway in 4T1 cells (related to Figure 3), and Figure S4 showing ES-CM inhibit tumor growth in vivo (related to Figure 5). (DOCX 4638 kb)
