# Supplementary material for: Embryonic stem cell preconditioned microenvironment suppresses tumorigenic properties in breast cancer
Source: Stem Cell Res Ther. 2016 Jul 27;7:95. doi: 10.1186/s13287-016-0360-x (PMC4962384; doi:10.1186/s13287-016-0360-x)
Supplement: Additional file 1: — Supplementary information contains Table S1 presenting primer sequences used for real-time PCR, Figure S1 showing mES cell characterization and reporter gene cells used in the experiment, Figure S2 showing decreased proliferation of 4T1 cells by direct cell–cell contact of ES cells, Figure S3 showing ES-CM inhibited Stat3 signaling pathway in 4T1 cells (related to Figure 3), and Figure S4 showing ES-CM inhibit tumor growth in vivo (related to Figure 5). (DOCX 4638 kb) [file 13287_2016_360_MOESM1_ESM.docx]

**Supplementary Information:**

**Embryonic Stem Cells Preconditioned Microenvironment Suppresses Tumorigenic Properties in Breast Cancer**

Ningning He, Guowei Feng, Yang Li, Yang Xu, Xiaoyan Xie, Hui Wang, Yuebing Wang, Lailiang Ou, Xuetao Pei, Na Liu, Zongjin Li

**Supplementary Table S1**

**Supplementary Figures 1-4**

**Table S1. Primer sequences used for real-time PCR**

| Gene | Forward primer (5’→3’) | Reverse primer (5’→3’) |
| --- | --- | --- |
| *Nanog* | GCTCCGCTCCATAACTTCG | ACCTGGCTTTGCCCTGACT |
| *Oct4* | CCCAACGAGAAGAGTATGAGG | GAGCAGTGACGGGAACAGA |
| *Sox2* | TGGACTGCGAACTGGAGAAG | ATTTGGATGGGATTGGTGGT |
| *Klf4* | CTCGGGCGGCTTCGTG | CGTCTGGGCTTCCTTTGC |
| *Stat3* | GCCATCCTAAGCACAAAGCC | GGGAATGTCGGGGTAGAGGT |
| *c-Myc* | GACTGTATGTGGAGCGGTTTCT | TGCTGTCGTTGAGCGGGTA |
| *β-catenin* | GTCTGGACTCGCCGCCTAT | TTGCGCCTCCGTTTGG |
| *Ang-1* | ACAGGGACAGCAGGCAAAC | GGCATCGAACCACCAACC |
| *Ang-2* | GACTGGGAAGGCAACGAG | CTGAGAGCATCTGGGAACA |
| *Hif-1α* | GTGCACCCTAACAAGCCGGGG | AGCACCAAGCACGTCATGGGT |
| *Pigf* | CTTCTGAGTCGCTGTAGTGG | TCCTTTCTGCCTTTGTCG |
| *Pdgf* | ATCCAGGGAGCAGCGAGCCA | CAGGGCCGCCTTGTCATGGG |
| *Sdf-1α* | CCAAGGTCGTCGCCGTGCTG | CTCGAAGAACCAGCAGGGGC |
| *N-cadherin* | ATGTGCCGGATAGCGGGAGC | TACACCGTGCCGTCCTCGTC |
| *Vimentin* | CTTGAACGGAAAGTGGAATCCT | GTCAGGCTTGGAAACGTCC |
| *Snail* | AAGATGCACATCCGAAGCCA | CTCTTGGTGCTTGTGGAGCA |
| *E-cadherin* | CACCTGGAGAGAGGCCATGT | TGGGAAACATGAGCAGCTCT |
| *Tgf-β1* | AACTATTGCTTCAGCTCCAGAGAGA | AGTTGGATGGTAGCCCTT G |
| *Gapdh* | GCACAGTCAAGGCCGAGAAT | GCCTTCTCCATGGTGGTGAA |

**Supplementary Figures:**

**
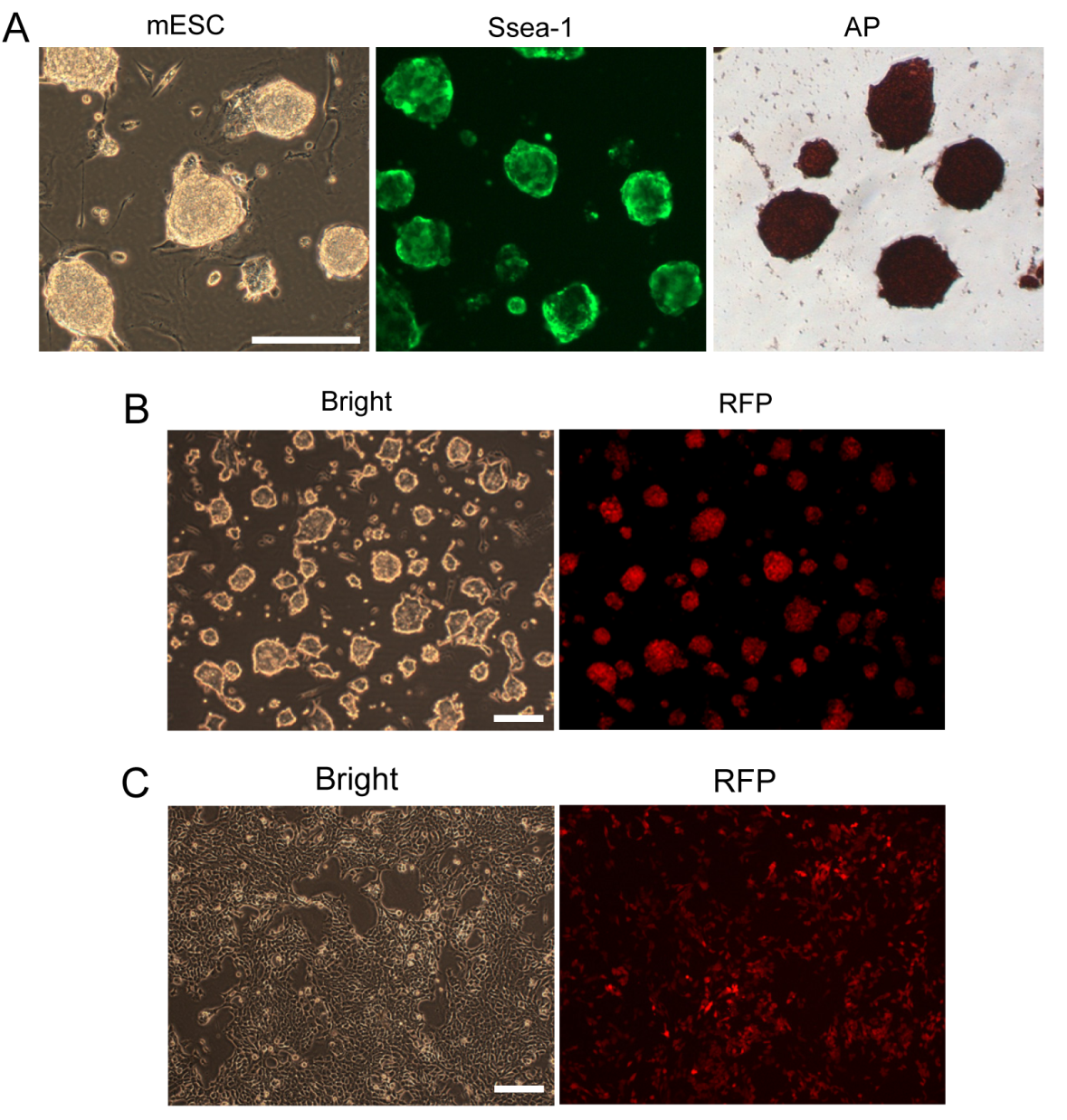
**

**Figure S1. Murine embryonic stem cells (mES cells) characterization and reporter gene cells used in the experiment.** (A) *In vitro* characterization of mES cells. Phase contrast microscope showed tightly aggregated and uniformly round ES cell colonies. All of the cells inside ES colonies were Ssea-1 and AP positive. The scale bar represents 100 μm. (B&C) Transduced mES and 4T1 cells are strongly positive for RFP on ﬂuorescence microscopy.

**
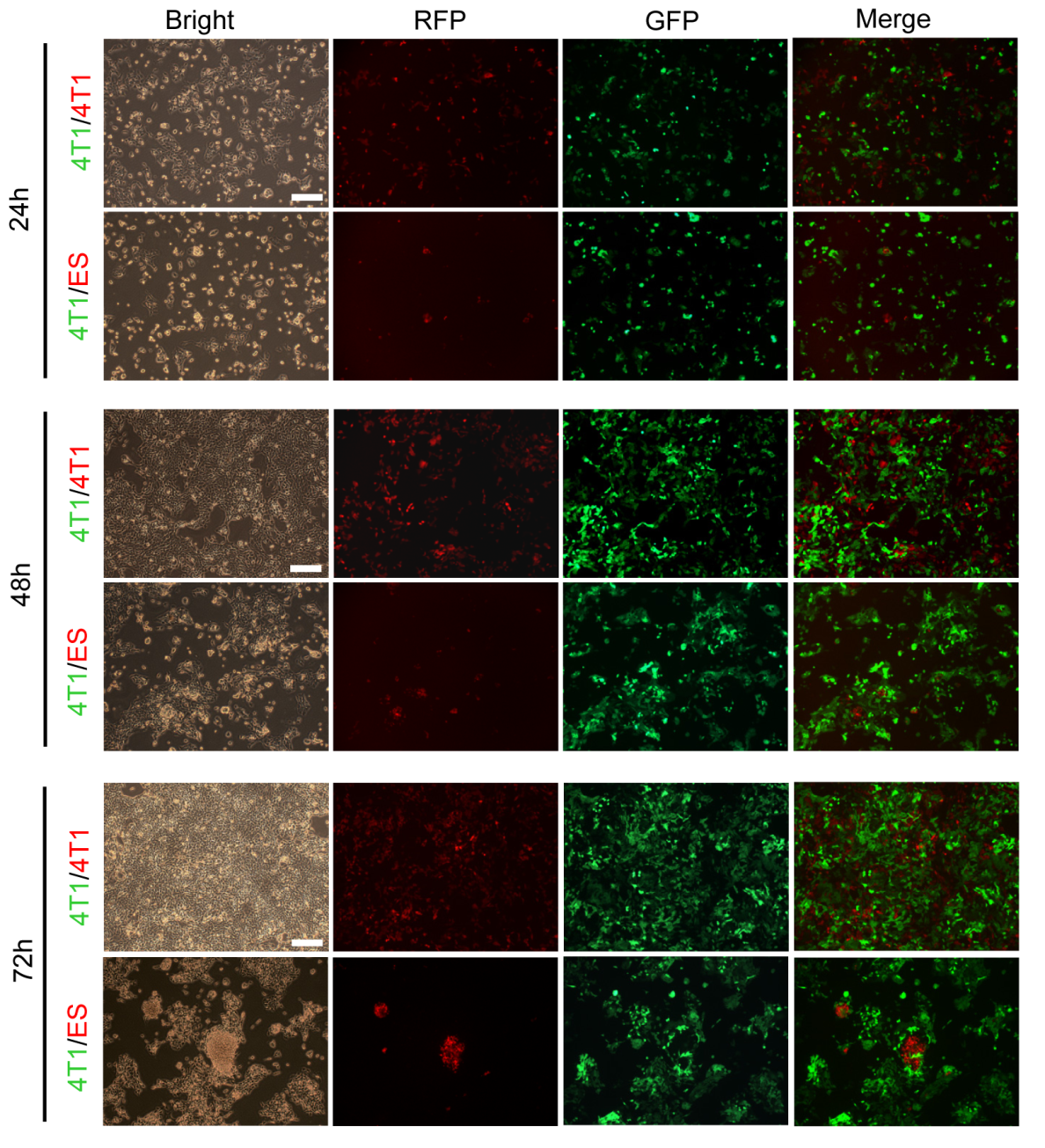
**

**Figure S2. Decreased proliferation of 4T1 cells by direct cell-cell contact of ES cells.**

**
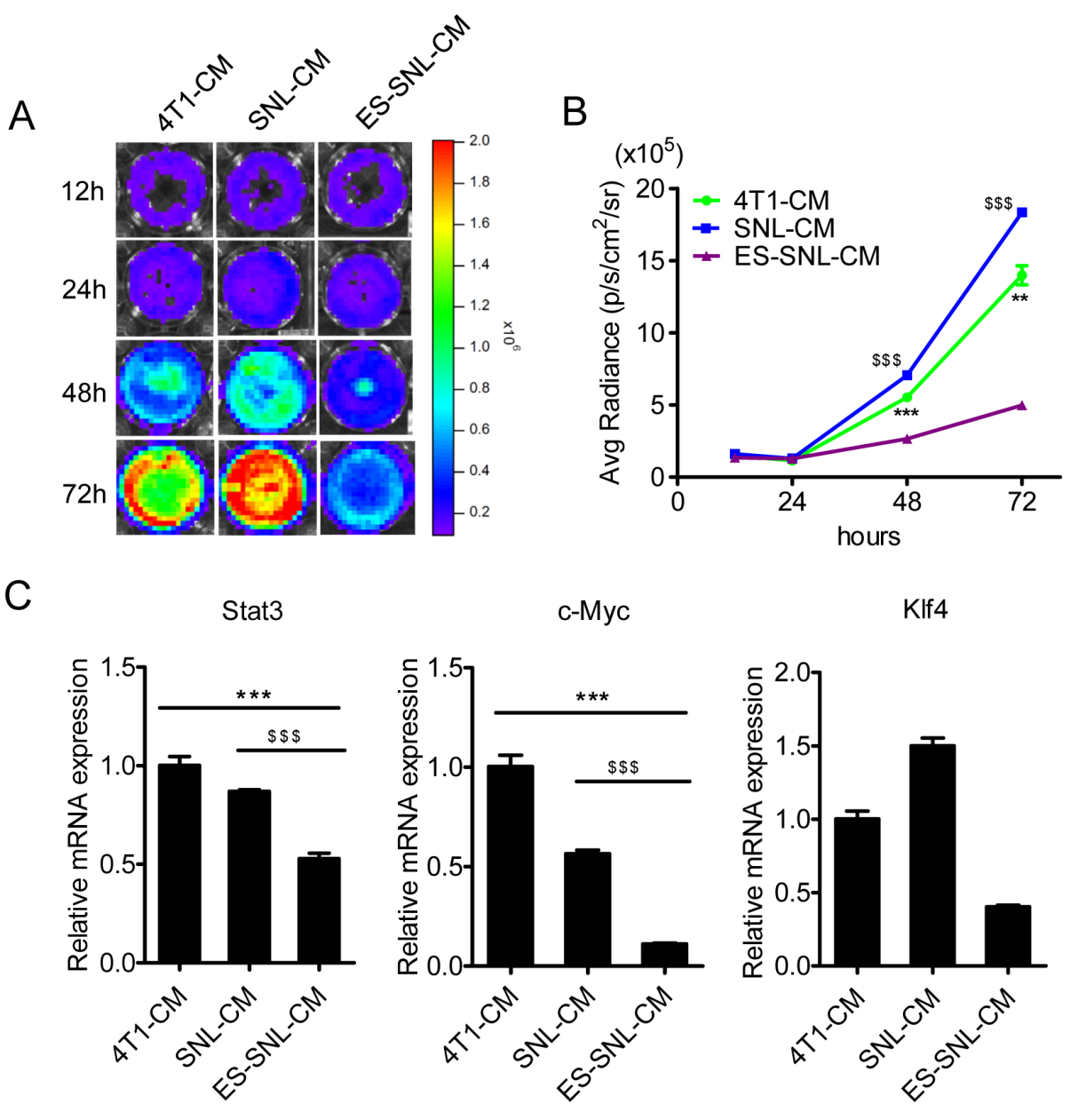
**

**Figure S3. ES-CM inhibited Stat3 signaling pathway in 4T1 cells, related to Figure 3.** (A) Renilla luciferase (Rluc) imaging of activated Stat3 *in vitro* controlled by 4T1-CM and SNL-CM. (B) Quantitative analysis of imaging signals. The signal activity showed the suppressed effect of ES-SNL-CM group. ***P*< 0.01 vs 4T1-CM, ^$$^*P*< 0.01 vs SNL, ****P*< 0.001 vs 4T1-CM, ^$$$^*P*<0.001 vs SNL-CM, n=3. (C) Analysis of Stat3 signaling pathway related genes expression．***P < 0.001 vs 4T1-CM，^$$$^*P*<0.001 vs SNL-CM, n=3.


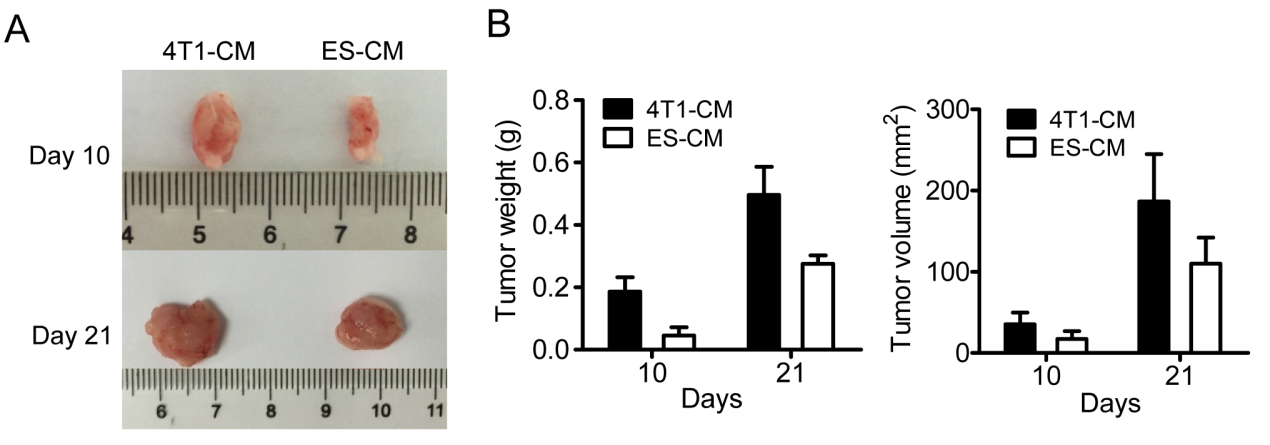


**Figure S4. ES conditioned medium inhibit tumor growth *in vivo,* related to Figure 5.** (A) The difference in tumor sizes at day 10 and 21 after cells injected in two groups, n=5. (B) Tumor volumes and weights were measured. n=5.
